# Microfluidic Device on Fused Silica for Raman Spectroscopy of Liquid Samples

**DOI:** 10.3390/bios15030172

**Published:** 2025-03-06

**Authors:** Celia Gómez-Galdós, Andrea Perez-Asensio, María Gabriela Fernández-Manteca, Borja García García, José Francisco Algorri, José Miguel López-Higuera, Luis Rodríguez-Cobo, Adolfo Cobo

**Affiliations:** 1 Photonics Engineering Group, Universidad de Cantabria, 39005 Santander, Spain; 2 Instituto de Investigación Sanitaria Valdecilla (IDIVAL), 39011 Santander, Spain; 3 CIBER de Bioingeniería, Biomateriales y Nanomedicina, Instituto de Salud Carlos III, 28029 Madrid, Spain

**Keywords:** microfluidic, ULAE, Raman spectroscopy, cyanobacteria, continuous flow

## Abstract

Water testing is becoming increasingly important due to dangerous phenomena such as Harmful Algal Blooms (HABs). Commonly, the content of a water sample is measured for the detection, monitoring and control of these events. Raman spectroscopy is a technique for the molecular characterization of materials in solid, liquid or gaseous form, which makes it an attractive method for analysing materials’ components. However, Raman scattering is a weak optical process and requires an accurate system for detection. In our work, we present, from design to fabrication, a microfluidic device on fused silica adapted to optimise the Raman spectrum of liquid samples when using a Raman probe. The device features a portable design for rapid on-site continuous flow measurements avoiding the use of large, costly and complex laboratory equipment. The main manufacturing technique used was ultrafast laser-assisted etching (ULAE). Finally, the effectiveness of the microfluidic device was demonstrated by comparing the Raman spectra of a known species of cyanobacteria with those obtained using other conventional substrates in laboratory analysis. The results demonstrate that the microfluidic device, under continuous flow conditions, exhibited a lower standard deviation of the Raman signal, reduced background noise and avoided signal variations caused by sample drying in static measurements.

## 1. Introduction

Harmful Algal Blooms (HABs) represent a critical environmental issue, characterized by the uncontrolled proliferation of aquatic organisms, particularly those that produce toxins. The toxins produced by these organisms are directly incorporated into the surrounding water, posing significant risks to both aquatic life and human health. Those who consume contaminated water or organisms are at risk of intoxication [1,2]. Therefore, the identification and quantification of harmful species are crucial for assessing the potential danger [3]. This issue has become a global concern, with research focusing on the detection, monitoring and removal methods of HABs [4]. Water treatment strategies depend on the type and concentration of the organisms present [5,6,7], making the detection and accurate identification of species essential. Traditional methods, such as taxonomic classification [8], aim to identify and quantify different phytoplankton species in both marine and freshwater environments [9,10]. Consequently, the prevention of HABs relies heavily on efficient monitoring of water quality.

Traditionally, HABs’ detection is performed by bringing water samples from the studied area to the laboratory, where expensive, bulky and heavy equipment is located. Conventional methods for liquid sample analysis are usually based on visual identification of cells, such as bright-field microscopy, scanning electron microscopy (SEM), transmission electron microscopy (TEM) or fluorescence in situ hybridization (FISH) [11,12]. FISH is a powerful tool to overcome resolution problems in microscopic assays. DNA or RNA probes are fluorescently labelled and bind to unique genetic sequences of target organisms, allowing for their visualization under a microscope [13]. Although these techniques provide high specificity, they are also very time consuming, both in terms of sample preparation and the measurement process. Typically, a counting chamber is used to assist with the quantification of aquatic organisms observed in the sample [14]. However, these methods are labour intensive and require highly trained personnel.

Meanwhile, other molecular and chemical techniques have emerged to identify toxin content in aquatic environments, such as polymerase chain reaction (PCR), enzyme-linked immunosorbent assay (ELISA) and microarray biosensors [15]. PCR is a widely used molecular technique to identify specific DNA sequences in a sample. The procedure starts with extracting DNA from the water sample, followed by amplification of the target DNA sequence using primers specific to the organism of interest. PCR allows for the rapid identification of species but requires careful sample preparation to prevent DNA degradation. The process typically takes 2–3 h, with amplification time depending on the size of the DNA fragment. Although PCR offers fast analysis, it primarily identifies species and does not provide direct information on toxin concentration or quantify the organisms present [16]. On the other side, ELISA allows for the detection and quantification of specific toxins or biomarkers in a water sample, such as microcystins or saxitoxins, using biochemical methods. The procedure involves adding a sample to wells pre-coated with specific antibodies, followed by the addition of the enzyme substrate. The antibodies bind to the toxins and produce a colour change when the substrate reacts. This method typically takes 2–6 h, depending on the assay, and requires careful sample preparation to ensure toxins remain intact. While ELISA is effective for toxin detection, it does not provide information about the species responsible for toxin production, limiting its ecological insight [17]. Lastly, microarrays perform multiple biochemical assays simultaneously. These biosensors consist of a solid surface (such as a chip or slide) onto which specific probes—typically nucleic acid sequences or antibodies—are attached. When water samples are applied to the microarray, the target molecules from the HAB organisms, such as toxins or genetic material, bind to the corresponding probes on the array. The interaction is detected through various techniques, with fluorescence or electrochemical signals commonly used [18,19,20]. Microarrays typically require 4–6 h for hybridization and signal detection. The method is suitable for detecting multiple targets simultaneously but still requires sample preparation, such as DNA/RNA isolation or toxin extraction, and does not directly quantify organisms or toxins in the sample [18,19,20]. While molecular methods, such as PCR and ELISA, offer faster analysis than traditional microscopy, they tend to be less specific in identifying the exact species involved and do not provide direct measurements of the organism’s concentration. These methods also do not allow for continuous monitoring and are not suitable for in situ or real-time analysis, which is a significant limitation for large-scale monitoring of water bodies. Additionally, both PCR and ELISA often require specialized equipment and reagents, which can be costly and time consuming to prepare.

Optical spectral analysis techniques have also been used to provide real-time results. These techniques allow for the classification of phytoplankton into spectral groups based on their fluorescence characteristics, enabling rapid data acquisition. However, fluorescence-based methods often require complementary techniques for accurate species identification, as fluorescence alone cannot provide definitive taxonomic information. Additionally, the use of fluorescent dyes or stains may require additional sample preparation steps, such as incubation times, which can vary between 30 min and 2 h, depending on the specific staining protocol [21,22,23]. While these techniques provide fast results, they are limited by their inability to distinguish between species with similar fluorescence profiles and are typically used for classification rather than precise identification.

In brief, while molecular and optical methods offer faster analysis times than traditional microscopy, they generally come with trade-offs in terms of specificity and real-time monitoring capabilities. The limitations of the current detection and monitoring devices present opportunities for developing a technological solution for analysing liquid samples. In this regard, Raman spectroscopy performs precise component identification of materials. A few studies have already been presented in this field for the identification of aquatic organisms in liquid samples from various ecosystems [24,25]. However, these techniques are still mostly confined to laboratory environments. To achieve more precise and reliable results, especially for in situ and real-time monitoring, the integration of advanced techniques into microfluidic devices offers significant promise [26,27], as in the case with Raman spectroscopy [28].

Raman spectroscopy is a powerful tool for accurately identifying the composition of materials in a non-invasive way. The Raman spectroscopy technique involves the energy shift between the incident light and the detected light, i.e., the result of the light–matter interaction corresponding to Raman scattering. This inelastic scattering is related to molecular vibrations within the material where the light interacts [29]. In fact, the Raman spectrum represents the intensity of the shifted light and is considered the molecular fingerprint of the material due to the peaks in the spectrum that identify different bonds in the molecular composition of the sample [30,31].

Confocal Raman microscopy has traditionally been used for the acquisition of Raman spectral data, where the analysis of the material is mainly limited to the components in the plane of focus [32]. Thus, the analysis of liquid sample is often performed on droplets that are left to dry on a sample holder, extending measuring times and leading to another problem commonly referred to as the “coffee-ring” effect: an uneven deposition of analytes that are mainly directed to the edge of the droplet [33]. Therefore, the measurements taken at the centre are different from those taken at the boundary of the droplet, which poses a problem in the analysis of the results. Whereas Raman spectroscopy used to be performed by confocal Raman microscopy equipment, using a Raman probe enables integration to a portable system for rapid on-site measurements of water quality. However, Raman probe measurements heat the sample, and in the case of a liquid sample when a deposited drop is under analysis, the sample is progressively dry. Thus, on biological samples, the latest measurements could represent different compositions from the initial ones. Then, a measurement system where the nature of the sample remains stable is necessary.

On the other hand, Raman scattering is a weak process. Statistically, when light interacts with matter, thousands of photons are scattered out of a million incident photons, and only one photon is due to inelastic Raman scattering. Therefore, techniques are often employed to improve the results of Raman spectroscopy. To enhance sensitivity, Surface-Enhanced Raman spectroscopy (SERS) is employed, which amplifies Raman signals by utilizing a roughened metal surface, making it particularly effective for detecting trace amounts of toxins and pollutants in water. For even greater enhancement, Tip-Enhanced Raman spectroscopy (TERS) combines Raman with atomic force microscopy (AFM), providing extremely localized analysis at the nanoscale. Additionally, Surface Selective Raman (SSR) spectroscopy is a technique used to analyse the surface layers of materials, offering unique insights into the interactions at interfaces in liquid systems. Together, these techniques provide a wide range of options for detecting and identifying substances in aquatic environments, from large-scale analysis to highly sensitive, localized measurements [24,34]. However, the objective of cost reduction led us to choose to prototype an inexpensive device adapted to enhance Raman probe measurements. Conclusively, a microfluidic device for Raman analysis of liquid samples in continuous flow using a probe was proposed.

In this work, we propose a microfluidic device designed for the analysis of liquid samples in continuous flow by Raman spectroscopy with a probe. Other typical substrates in laboratory analysis were used to validate the device by comparing Raman spectra results of a known sample. Specifically, the flow condition was compared with a static case using a counting chamber and wells, and a commercial microfluidic channel was considered to assess the suitability of the proposed device for Raman probe measurements. In addition, details of the design, manufacturing and validation methods used are presented.

## 2. Materials and Methods

### 2.1. Raman Spectroscopy Conditions

The key factor for the design of the microfluidic device for Raman spectroscopy analysis was the optimization of the sample–light interaction volume. In this regard, various parameters were considered: the Raman probe configuration, outer dimensions and beam shape. The configuration of the Raman probe to be used (532 nm RamanProbe^TM^, Inphotonics (Norwood, EEUU)) includes the following specifications: a focal length of 5 mm, a depth of field of 1.5 mm and a probe diameter of 9.52 mm. Additionally, the beam shape at the focal point is approximately 200 µm in spot diameter. These dimensional specifications were followed to adapt the microchannel (see Section 2.2).

### 2.2. Design

The aim of the design was to fabricate a microfluidic channel adapted to be integrated into a portable Raman probe system and to optimise the captured signal.

The material of choice for the device to perform Raman spectroscopy analysis was fused silica, taking advantage of the fact that there is no interference of this material with the Raman spectra of the sample under analysis. The microfluidic channel was then chosen to be fabricated on a 20×20×1 mm fused silica glass (FOCtek, ±0.1 mm size tolerance and ±0.01 thickness tolerance). Therefore, a curved shape for the channel was chosen to facilitate the integration of the microfluidic device with the Raman probe setup. Aimed at optimizing the measurement process and ensuring efficient analysis of the liquid sample, the channel was designed with a width of 200 µm to match the size of the beam of the Raman probe, thus fitting the sample region. The ends of the microchannel were widened and funnel shaped to facilitate access for the liquid sample. Thus, the 200 µm wide channel was progressively enlarged to connect it to a 1.5 mm diameter semicircle. As a thorough glass channel simplified the fabrication procedure and the focal volume of the Raman probe allowed a depth greater than 1 mm, the channel was designed through the glass. Thus, the glass allocating the channel was covered on both sides: a pristine glass and another identical glass with two access points at the ends of the microchannel. These points were intended for inserting tube adapters, with a 1.5 mm inside diameter to a selected tube diameter on the outside; in this case, the tubing has a 1/3 mm inside/outside diameter (silicon tubing k70, FHX006, from Kartell (Noviglio, Italy)) was used.

**Figure 1:**
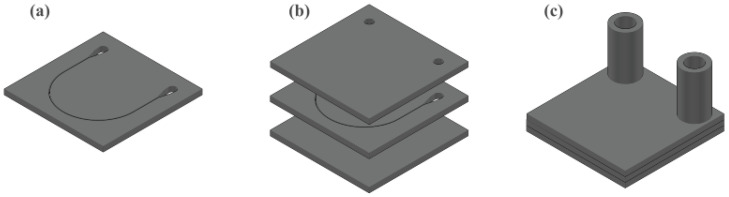
Three-dimensional view of the proposed design: (**a**–**c**) show the complete assembly process. (**a**) CAD design of the 20×20×1 mm glass with the through microchannel. (**b**) Glasses involved in the whole device. (**c**) Final appearance of the microfluidic device: overlapping glasses and tube adapters.

**Figure 2:**
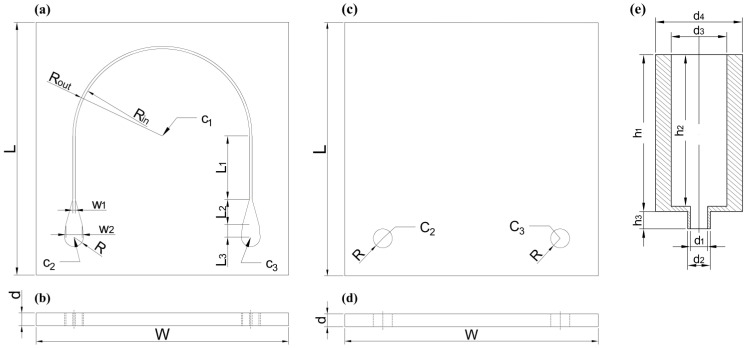
(**a**) Top view and (**b**) side view of the through microchannel design. (**c**) Top view and (**d**) side view of the covering with the access holes. (**e**) Cross-section of the tube-to-channel adapters. The labels of the design parameters are included and the values are shown in Table 1.

In brief, the device consists of a three-glass assembly coupled with the adapter pieces: an unmodified glass, at the bottom; a glass with the shape of the microfluidic channel inscribed through the glass, in the middle; and a glass with two 1.5 mm diameter holes at the ends of the microfluidic channel, at the top. These considerations are indicated in Figure 1 and the particular parameters for the manufacturing parts are shown in Figure 2 with the specification values in Table 1.

The key parameters of the design of the channel in the 20 × 20 × 1 mm (W × L × d) fused silica glass are as follows: w_1_ = 0.2 mm, the width of the channel matching the spot size of the Raman probe’s beam, and d = 1 mm, the depth of the channel aligns with the depth of focus of the probe, thus optimizing the measurement process to ensure efficient analysis of the liquid sample. The channel was designed with a curved shape between R_*out*_ = 7.10 mm and R_*in*_ = 7.10 mm, ensuring proper positioning of the Raman probe.

### 2.3. Fabrication

**Figure 3:**
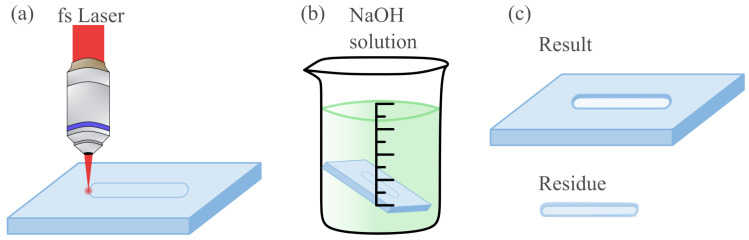
Schematic of the ULAE process applied to the designs: (**a**) first step, laser inscription in the glass; (**b**) second step, assisted wet etching; and (**c**) results.

Ultrafast laser-assisted etching (ULAE) was considered as the fabrication method due to its micrometer accuracy for the fabrication of the microchannel and holes in fused silica glass. ULAE is a two-step subtractive technique (Figure 3). First, the fused silica glass is irradiated by femtosecond laser pulses and then it undergoes wet chemical etching [35]. These are complementary processes, as the material is initially modified at highly localized points corresponding to the irradiation of laser focal points, and then the chemical solution affects the laser-modified areas more intensely than non-modified regions, by a factor of three orders of magnitude. The femtosecond inscription process is highly dependent on the polarization of the laser beam, unless under specific conditions, precisely termed the polarisation-independent region as determined by Ochoa, M. et al. [36]. Although the ULAE method started using HF as the chemical solution in the second step, nowadays, chemical bases such as NaOH are becoming more common. Etching rates with NaOH indicate 300 µm/h in damaged regions and 0.5 µm/h in pristine fused silica [36].

The inscription setup consists of a commercial femtosecond laser chirped pulse amplifier (FLCPA), Cazadero Series of CALMAR, from which pulses are precisely focused through an aspheric lens (A110TM-B, Thorlabs) with an NA =0.4 into the glass. The glass sample is placed on a nano-resolution XYZ-stage (A-3200, Aerotech (Pittsburgh, PA, USA, EEUU)). Finally, a CCD camera (DMK 31AG03, Imaging Source (Bremen, Germany)) is integrated to visualise the femtosecond laser beam on the glass by reflection, which is essential for the initial positioning of the centre of the glass. The laser source operates at a wavelength of 1030 nm and pulse duration of 370 fs, with a maximum pulse energy of 5 mJ and pulse repetition rates (PRRs) up to 480 kHz. The described system leads to micrometre accuracy (±2 µm) on inscriptions.

As mentioned earlier, the fabrication of the microfluidic device involved a fused silica glass with a through channel that was covered on both sides: one side with an identical glass featuring access points, including an inlet and outlet for sample insertion, placed at the extreme positions of the microchannel, and the other side with an identical pristine glass. Accordingly, the ULAE process was applied on two different glasses. In this case, both glasses were inscribed under polarisation-independent conditions. The through channel was inscribed with a PRR of 3 kHz and an energy of 0.80 µJ, while for the holes, a PRR of 12 kHz and an energy of 0.78 µJ were applied. Both inscriptions were also etched for three hours in NaOH (from ITW Reagents, Sodium Hydroxide pellets, reference number: 131,687.1211) (5 wt% solution) maintained at 85±2 °C and constantly stirred at 500 rpm. Once removed from the solution, the glasses were sonicated and then cleaned with distilled water to completely remove any glass debris.

The tube-to-channel adapters of the microfluidic device were fabricated in polymer using an additive manufacturing technique: they were printed on a Form 3B printer (ColorfulQuoll serial from Formlabs) using draft resin V2 with an accuracy of ±100 µm. After printing, the pieces were washed for 20 min and cured for 60 min at 60 °C (using a Form Wash containing isopropanol and a Form Cure, from Formlabs, respectively).

**Figure 4:**
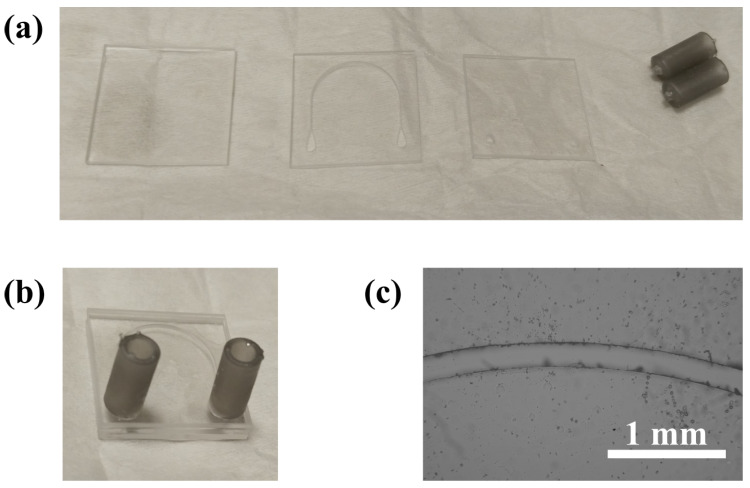
Initial (**a**) and final (**b**) images showing the assembly process of the device in the laboratory. (**a**) The three glasses and the two resin adapters before they are glued together, and (**b**) is the result after adhesion. (**c**) Microscopic image of the microchannel at the central point of the curve, captured after the manufacturing process.

Finally, the inscription with the holes and a pristine glass were glued by covering the glass with the microchannel inscription using optical adhesive (NOA61, from Thorlabs (Puteaux, France)) and UV curing (High-Power UV Curing LED System, from Thorlabs). An important issue during bonding was ensuring that the channel remained free of adhesive. The same consideration was applied for the gluing of the adapters to the two glass access points. Then, in the glass bonding process, three drops of adhesive were placed at intermediate points between the channel and the edges of the glasses. Upon applying pressure to both glass pieces, the adhesive was evenly distributed between the two surfaces, extending to the edges of both the glass and the channel. At the edges of the glass, the adhesive was spread into a thin layer to further secure the bond. Within the channel, it was necessary to clean the excess adhesive to prevent it from becoming a permanent obstruction after curing the adhesive. To achieve this, with both glass pieces pressed together, acetone was circulated through the channel to remove the excess adhesive. Acting as a solvent, the acetone carried the adhesive towards the outlet. Subsequently, distilled water was circulated to remove the acetone and prevent its capillary action from causing it to seep between the glass surfaces and dissolve some of the adhesive. Finally, the curing process was performed to permanently secure the glass pieces. The process that was described in the schematic of Figure 1 is shown in the laboratory work in Figure 4.

### 2.4. Microfluidic Device in Operation

**Figure 5:**
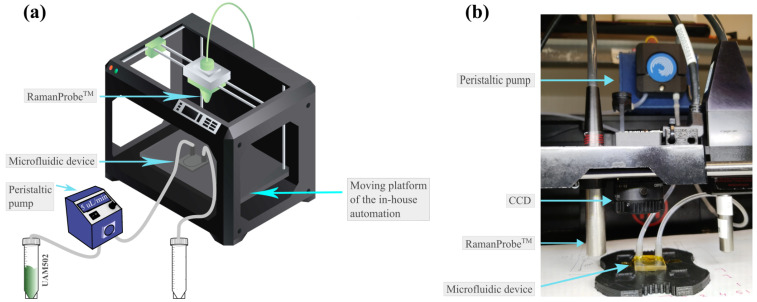
(**a**) Schematic diagram of the assembly corresponding to the integration of the microfluidic device in the RamanProbe equipment. (**b**) Image during operation of the microfluidic device in RamanProbe measurements.

The operation of the microfluidic device required the installation of tubing into the resin parts to create a circuit for the sample flow through the device. The inlet tubing was connected to the outlet of an open-source peristaltic pump, and the outlet tubing was directed to a collection container. The input tube of the peristaltic pump corresponded to the liquid sample container. Figure 5a shows the above configuration, as well as the placement of the device in the Raman spectroscopy system. Raman measurements were performed using a custom Raman probe setup from Inphotonics, which employs a 532 nm RGB Lasersystems Lambda Laser and QEPRO Ocean Insight’s spectrometer. The equipment was integrated into an in-house automation system, where a CCD camera was used to target the measurements, and data acquisition and processing were performed using software based on Matlab (R2024a). Finally, an image of the complete unit in the laboratory is shown in Figure 5b.

### 2.5. Validation Criteria of the Microfluidic Device for Raman Spectroscopy Technique

The fabricated device, a microfluidic channel with dimensions adjusted to the optimisation of RamanProbe^TM^ measurements, was validated by comparing the Raman spectra of a liquid sample with a known composition (Section 2.5.1) with respect to other substrates (Section 2.5.2) and under identical conditions (Section 2.5.3). After processing the spectra, as explained in Section 2.5.3, the analysis of the results for microfluidic device validation was carried out by evaluating the measured Raman signal quality and comparing the results obtained from the different substrates. The validation criteria were based on the ease of identification of the reference sample in the spectrum and the standard deviation of the recorded spectra.

#### 2.5.1. Reference Sample

The reference liquid sample for Raman analysis was an extract from a pure laboratory culture of the cyanobacteria species *Dolichospermum crassum* UAM502. The sample was obtained from and provided by the Department of Biology at the Autonomous University of Madrid (UAM). The specimen was maintained in BG11_0_ medium, a standard, nutrient-rich medium for cyanobacterial cultivation, at 25 °C and illuminated for 24 h with light intensity between 70 and 130 µmol photons m^−2^s^−1^. To ensure optimal growth conditions, the cultures were regularly agitated by hand to prevent cell sedimentation and facilitate gas exchange.

The concentration of the test sample was 866 cell/µL, calculated using the same counting chamber considered as a substrate in the validation process.The subsamples considered for each measurement in the study remained unaltered compared to the standard. This cyanobacteria species can be found in real water samples from different aquatic environments. Therefore, this reference standard is considered for initial validation and serves as a first step towards the application of the microfluidic device to real samples.

The Raman spectrum of the sample should exhibit three main peaks, associated with carotenoid compounds, with shifts at 1002 cm^−1^, 1155 cm^−1^ and 1520 cm^−1^ [37].

#### 2.5.2. Considered Substrates

The fabricated device was compared with other conventional substrates for microscopic assays and content classification/quantification in liquid samples: the counting chamber and some custom wells. While these cases refer to a static situation of the sample on the substrate, the presented device was designed for the analysis under dynamic conditions. A commercial flow chamber was also considered. Therefore, the validation of the designed microfluidic device was performed by comparing it with the use of such elements as substrates when using Raman probe mounting for the analysis of the sample of reference (Section 2.5.1).

**Figure 6:**
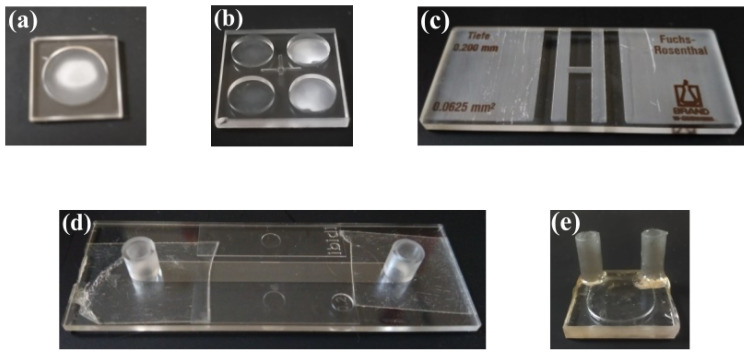
Substrates considered in the device validation process: (**a**) 20 µL single-well plate, (**b**) 5 µL 2 × 2 well array plate, (**c**) counting chamber, (**d**) µ-Slice I^0.2^ Luer and (**e**) the microfluidic device.

The measurements were performed by analysing the same amount of each sample: 20 µL. On the substrates related to the static situation, the counting chamber and the wells, a drop was deposited. The counting chamber used was the Fuchs-Rosenthal BRAND^TM^ Blaubrand^TM^ (BRAND^®^, Wertheim, Germany), with a $16\;{\mathrm{mm}}^{2}$ area delimited and regularly divided into 16 regions of $1\;{\mathrm{mm}}^{2}$ each, which are further subdivided into 16 equal areas of $0.0625\;{\mathrm{mm}}^{2}$. The 20 µL sample drop in the chamber covered a region of 10 mm diameter, approximately. The well plates were custom fabricated using the ULAE technique in two fused silica glasses of size 10×10×1 mm (fused silica window, from FOCtek (Fujian, China)) with two different configurations: a single well with a depth of 0.5mm and diameter of 7.2mm, and a 2 × 2 array of wells each with a depth of 0.5mm and a diameter of 3.6mm, equally distributed on the glass. The volume of the well in the single-well plate was exactly 20 µL, while in the plate with a set of wells, each well has a volume of 5 µL, and all wells were measured together. For continuous flow Raman measurements, the commercial flow chamber µ-Slice I^0.2^ Luer Uncoated (from IBIDI (Münich, Germany)) was used, along with the proposed device. The Luer chamber has a channel size of 5×35 mm and a depth of 0.2 mm, while the proposed microfluidic device features a channel with a width of 0.2 mm and a depth of 1.0 mm, extending over a path of approximately 33 mm. The defined substrates are shown in Figure 6.

The results presented mainly focus on the analysis of the spectra obtained from the sample under study, using the following substrates: a 20 µL single-well plate, a 5 µL 2 × 2 well array plate, a Fuchs-Rosenthal chamber, a µ-Slice I^0.2^ Luer and the microfluidic device. These substrates were selected as references for the following reasons: the counting chamber was evaluated for its versatility, allowing it to be transferred from the microscope to the Raman system after visual analysis; custom wells fabricated from the same material as the device under validation were used to compare measurements in static versus dynamic conditions; the two different well configurations were examined to assess whether varying droplet sizes affect Raman probe measurements as the droplet dries; and the commercial flow chamber was considered to highlight the advantages of the suited microfluidic device over existing tools.

#### 2.5.3. Raman Spectroscopy Measurements

First, Raman spectroscopy was performed for all the considered substrates, 20 µL single-well plate, 5 µL 2 × 2 array well plate, Fuchs-Rosenthal chamber, µ-Slice I^0.2^ Luer and microfluidic device, prior to sample deposition or flow, to consider substrate interference (see Section 2.5.2). Next, Raman spectroscopic studies of the UAM502 sample required two different procedures: one for the static and another for the dynamic situation. In the static case, pipetting was used, while in the dynamic case, flow was applied. For the static situation, a 20 µL sample drop was pipetted (using autoclavable micropipette of adjustable volume 10–100 µL, Digipette MGB005) into the counting chamber and onto the single-well plate, while four 5 µL sample drops were pipetted into the set of wells. These static cases were analysed using the custom configuration of the Raman probe mounting for spatial mapping, with Raman measurements taken at different positions over the drops. For the dynamic situation, the Luer slice or the microfluidic device was connected to the flow system, linking a sample container to a waste container via the peristaltic pump mentioned in the section about the microfluidic device in operation (Section 2.4). Additionally, both the Luer and the microfluidic device allowed for a fixed configuration of the Raman probe.

In general, the acquisition of Raman spectra was performed under identical conditions of integration time: 0.48 s, and laser power: 25 mW. In addition, all the substrates in the static situation were mapped by a 0.2 mm step, to fully utilise the laser spot size. For the substrates in the dynamic case, the flow rate was adjusted to mimic the 0.2 mm step size used in the mapping. Accordingly, a sample pump speed of 5 µL/min was selected. The dynamic case also required determining the number of measurements needed to analyse 20 µL of sample flowing through each substrate, 2500 measurements in the Luer analysis and 500 for the microfluidic device analysis, due to differences in depth.

**Figure 7:**
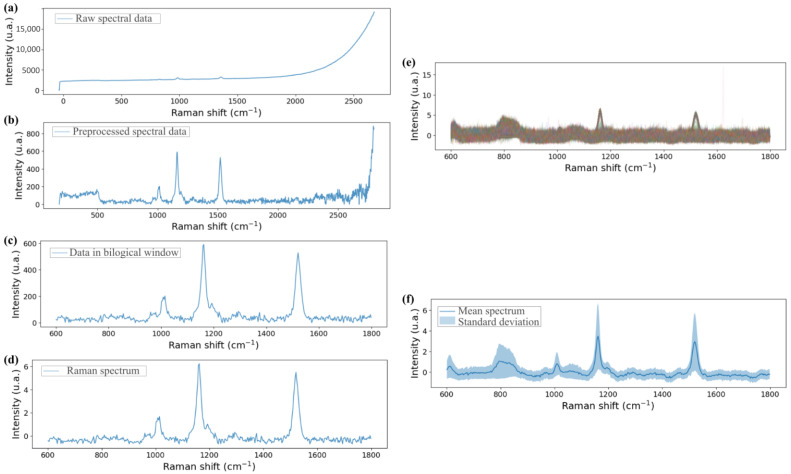
Scheme of data processing: from (**a**) raw spectrum to (**b**) preprocessed step with baseline consideration, domain restriction and filtering. Then, (**c**) spectrum was restricted to the ROI and (**d**) standardised. Finally, from (**e**), each spectrum data set of each substrate, (**f**) the mean spectrum and standard deviation were considered.

Once the measurements were completed, the raw Raman probe data were processed as outlined in the schematic in Figure 7. First, a baseline correction was applied to each spectrum, and the measurements were limited from 250 cm^−1^ to 2750 cm^−1^ to avoid saturation and minimise error propagation before filtering. Next, the spectrum was focused on the region of interest (ROI), which, for our work using cyanobacteria species as the reference sample, was the biological window, spanning from 600 cm^−1^ to 1800 cm^−1^. Finally, the spectrum-to-spectrum data were standardised, and the data set was averaged, with the standard deviation taken into account. Notice that the described process was applied separately to the spectra of each substrate.

Subsequently, the results were analysed. The microfluidic device was validated by evaluating the quality of the Raman signal obtained from the different substrates and comparing the outcome to those of the device. Three key aspects were considered in this analysis: first, the Raman signal of the substrate itself; second, the ease of identifying the reference sample within the processed Raman spectrum; and third, the standard deviation of the Raman spectra acquired during the measurements for each substrate. The standard deviation serves as an indicator of the consistency of the Raman signal during data acquisition, reflecting the uniformity of the measurements across the entire sample volume. Thus, the standard deviation represents the accuracy and reproducibility of the results. By integrating these factors, a comprehensive evaluation of the performance of the microfluidic device was achieved.

## 3. Results

Hereafter, in all Raman spectrum plots, three grey bands are shaded, corresponding to the areas where the peaks of the Raman signal of the reference sample should be found: with centres at 1007, 1160 and 1520 cm^−1^ and a width of 20 cm^−1^.

**Figure 8:**
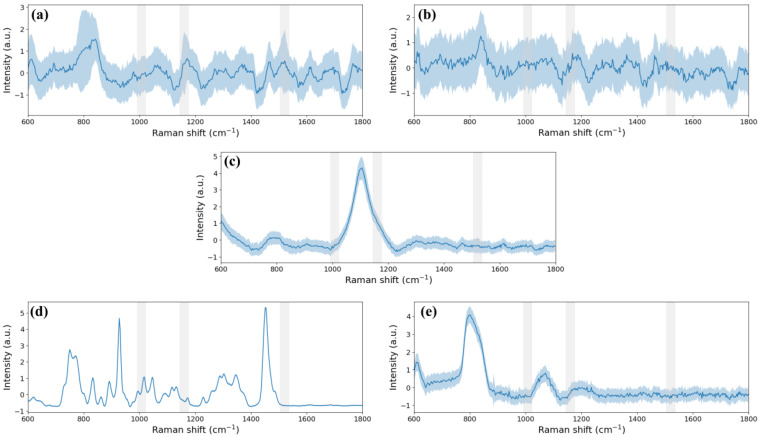
Mean Raman spectrum (line) and standard deviation (shadow) of the substrates: (**a**) 20 µL well, (**b**) 5 µL wells and (**c**) counting chamber, (**d**) Luer µ-Slide and (**e**) microfluidic device. Three grey bands indicating the position of the peaks of the reference sample are included.

Before measuring the reference sample, the Raman signal of the considered substrates, 20 µL single-well plate, 5 µL 2 × 2 well array plate, Fuchs-Rosenthal chamber, µ-Slice I^0.2^ Luer and microfluidic device, was evaluated. The measurement procedure and data processing were the same as for the analysis of the liquid sample but without sample deposition in the static cases or flow in the dynamic cases. The normalised mean spectrum and its respective standard deviation are presented in Figure 8. As the two plates of wells and the microfluidic device are based on fused silica glass, similar results were expected. Meanwhile, the 5 µL well provided a noisy signal, whereas the 20 µL well’s mean spectrum was smoother due to the use of a single ROI when measuring in the 20 µL well, compared to the utilisation of four ROIs when using the 5 µL wells. An identical standard deviation range is related to the material and fabrication process similarities. On the other side, there were sustainable differences between wells and the microfluidic device: a lower standard deviation range for the microfluidic device than the wells and the signals of the wells became more pronounced peaks when analysing the microfluidic device. This fact is related to the difference in the measuring process between the static and the dynamic situation. However, the results were very different from the results of the counting chamber and the Luer. In particular, the Raman signal of the Fuchs-Rosenthal chamber showed an intense, wide peak at 1100 cm^−1^, between the first two bands of interest for the reference sample. While the Luer exhibited several intense narrow peaks, these appeared outside the expected positions for the Raman signal of the reference sample. However, weaker peaks also appeared on the positions of the expected bands. This Raman signal from the Luer is primarily associated with the polymer composition of the microfluidic chamber.

**Figure 9:**
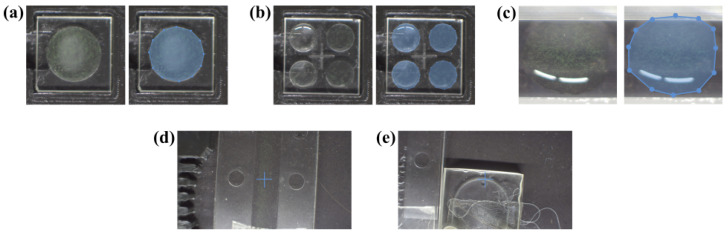
Photos of the substrates with a drop of sample deposited and mapping region: (**a**) 20 µL well, (**b**) 5 µL wells and (**c**) counting chamber, or with sample flowing and measurement point indication: (**d**) Luer µ-Slide and (**e**) microfluidic device. All the photos were taken by the camera in the Raman probe mounting.

The reference sample (UAM502) was then either deposited on the substrates in the static situation or circulated through the channels in the dynamic situation. Before measuring, the camera integrated into the custom setup was used to take photos of the substrate–sample configuration in each case (Figure 9). The purpose of the images was especially related to the identification of the measured points. In this sense, for the static cases, the photos are duplicated to indicate the region where the 0.2 mm step-size mapping was performed, while in the dynamic cases, the photos are shown just once with the specification of the local point where all measurements were performed, while the liquid sample was flowing at 5 µL/min. The point was exactly the centre of the image, which is the middle of the drawn cross.

**Figure 10:**
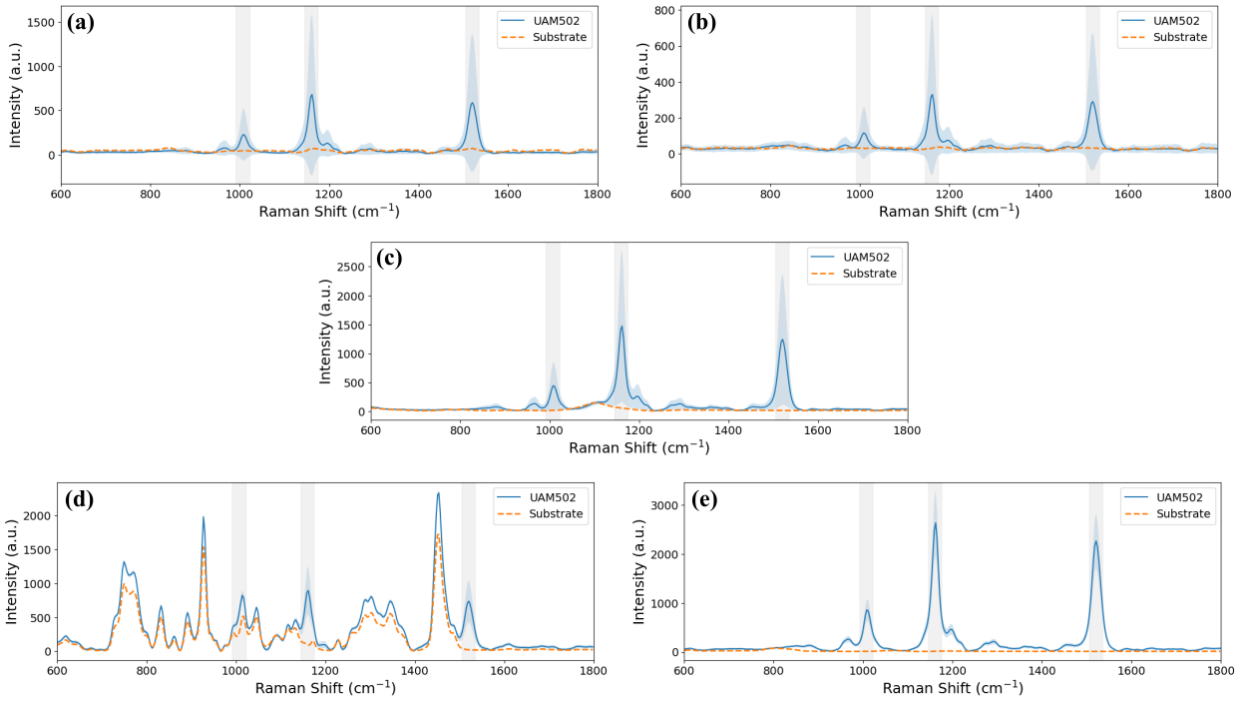
Mean Raman spectrum without normalisation of 20 µL of the reference sample (blue continuous line) and the substrate (orange dashed line) with standard deviation (shadow line on both sides) of the measurements when droplet deposition: (**a**) 20 µL well, (**b**) 5 µL wells and (**c**) counting chamber, or with sample flowing: (**d**) Luer µ-Slide and (**e**) microfluidic device. Three grey bands indicating the position of the peaks of the reference sample are included.

Then, the reference sample was measured in each case. The data obtained for each substrate with and without sample are represented in Figure 10. They were processed as described in Section 2.5.3 skipping the standardization step to correctly compare the two separately measured substrates. The shape of the reference sample spectrum was identical in all cases except the Luer µ-slide where the shape of the sample measurement was highly similar to that of the microfluidic chamber itself. However, when the substrate was one of those from the static cases, the standard deviation was large. In terms of intensity in the static cases, the captured Raman signal was lower in the 5 µL wells than in the 20 µL well and the counting chamber showed the highest peaks. However, the overlap of the mean spectrum of the counting chamber and the mean spectrum of the UAM502 drop in this substrate showed that the material alters the Raman signal of the sample. Meanwhile, the Raman spectrum when the sample was in the Luer µ-slide presented a low standard deviation, but the UAM502 fingerprint was masked by the commercial chamber itself, as was also expected from the mean spectrum of the Luer µ-slide substrate. Hence, the spectrum where the peaks were most clearly identified was the one obtained when the sample was measured in the microfluidic device. Low background noise was observed in this case, as expected from the sample-free measurements, and a small standard deviation of the mean spectrum was obtained.

**Figure 11:**
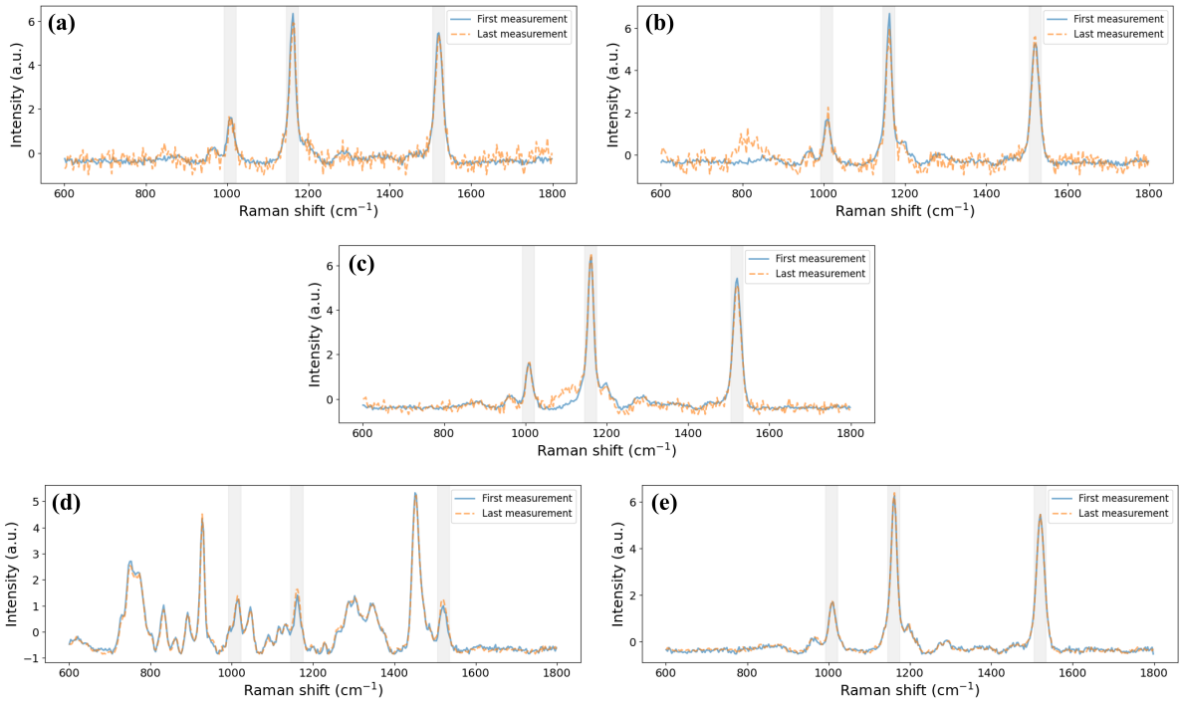
First (blue continuous line) and last (orange dashed line) Raman spectrum of 20 µL of the reference sample when droplet deposition: (**a**) 20 µL well, (**b**) 5 µL wells and (**c**) counting chamber, or with sample flowing: (**d**) Luer µ-Slide and (**e**) microfluidic device. Three grey bands indicating the position of the peaks of the reference sample are included.

Additionally, during Raman spectra acquisition in the static cases, it was observed that the drop dried up at the end of the measurement process and that the sample state changes affected the Raman signal. For this reason, the first and last Raman spectra of the reference sample when using each substrate were represented together (Figure 11). In the static cases, the last Raman spectrum was a noisier signal than the first Raman spectrum. Moreover, an analysis without normalisation showed lower intensity in the last measurement compared to the first. Therefore, there are significant differences between the spectra in all the static cases, while in the dynamic cases, the Luer µ-slide and the presented device, the spectra show nearly overlapping signals. This could be related to the standard deviation ranges in each case: significantly lower in the dynamic cases than in the static ones.

**Figure 12:**
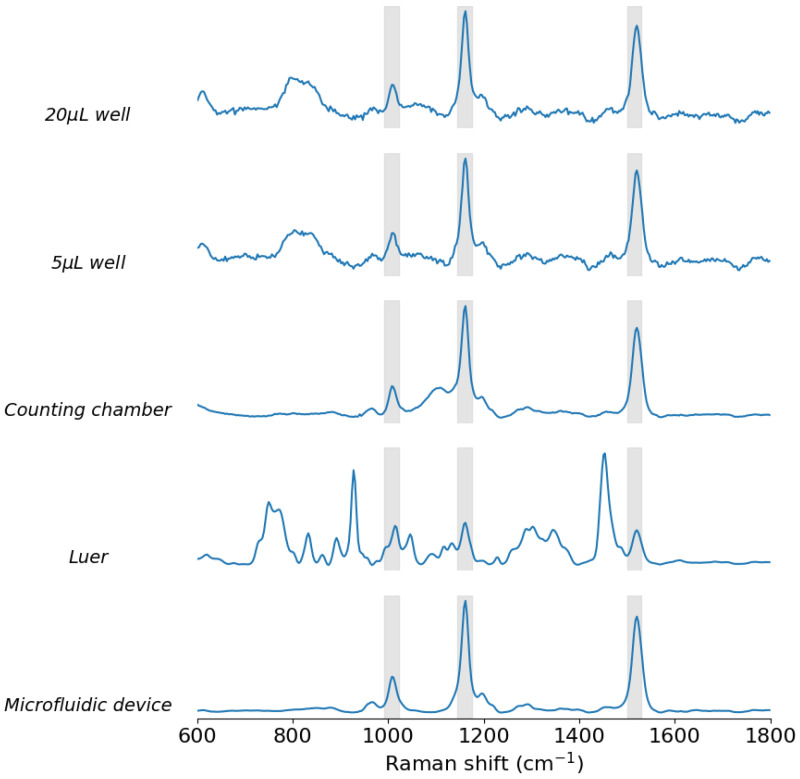
Mean spectrum of the spectra data set of 20 µL of the reference liquid sample measured: in the 20 µL well, in the 5 µL wells, in the counting chamber, in the Luer µ-slide and in the microfluidic device. Three grey bands indicating the position of the peaks of the reference sample are included.

Conclusively, the mean spectrum from all the analysed substrates, excluding the standard deviation, was plotted in Figure 12 for a clear qualitative comparison of the Raman spectra of the reference sample when using the different substrates and associated measurement techniques. According to all the previous analysis, all the reference substrates are appropriate except for the Luer µ-slide. Then, the counting chamber can be used to move a sample from a microscope system to the Raman setup; the different-sized wells gave similar results but these were more intense with a 20 µL volume than in 5 µL. Lastly, the microfluidic device exhibited the advantages of continuous flow measurements without the disadvantage of high Raman signal interference as seen with the commercial reference flow chamber.

Finally, the minimum detection limit of the microfluidic device was evaluated. To achieve this, the measurement of sequential dilutions from the reference sample as solute, and using the medium as solvent, provided a limit of detection (LOD) of 7 cell/µL.

## 4. Discussion

The corresponding measurement process for each substrate is summarised in Table 2. Sample preparation and system setup for measurement took more time and effort for static substrates. In particular, the configuration required in the dynamic cases was significantly easier compared to the static cases, while the results provided were also more stable, in terms of a lower standard deviation. Additionally, the measurement process itself was faster for the dynamic systems. In particular, the optimised design of the microfluidic device reduced the measurement time.

The microfluidic device is a portable system capable of acquiring real-time measurements of a liquid sample, operating on site and reducing both human and technological cost, and offering an advantage over conventional measurement systems. Additionally, it does not require sample preparation, and the results are obtained quickly after processing the collected signal. Furthermore, the system demonstrates excellent stability, as evidenced by the low standard deviation measured. One of the strengths of the microfluidic device is the appropriate selection of material. As shown, the reference substrate of the commercial flow chamber masked the Raman signal of the reference sample due to its composition. Reusability is another advantage of the microfluidic device. A cleaning procedure was required between measurements: circulating distilled water, a soap solution, ethanol (70%) and distilled water again, in intervals of two minutes at a flow rate of 15 µL/min. After this process, the Raman signal inside the channel became a noisy baseline, indicating that the device was ready for the next liquid sample.

Consequently, the feasibility of the device has already been demonstrated for the optimisation of liquid sample measurements using the Raman probe; the validation criteria using substrates for static measurements have also assessed the microfluidic device’s superiority, as mentioned in the Introduction [28]. On the other hand, a relatively high limit of detection was observed for the reference sample when compared to the minimum detection limits of other methods. Furthermore, while the microfluidic device is capable of composition identification of liquid samples, further studies are required to develop a method for content quantification. Therefore, additional research should be conducted to fully assess the capabilities of the device, including quantification or sorting capabilities. The aim of future work will be to apply the microfluidic device to real liquid sample analysis. Subsequent studies should focus on analysing different pure culture liquid samples, including limit of detection estimations, followed by the analysis of mixtures.

## 5. Conclusions

The presented device is a microfluidic channel technology solution optimised for Raman probe integration for content analysis of liquid samples. The functionality of the device was validated by comparing its Raman spectra of cyanobacteria with those obtained when the sample was deposited in different wells or in a counting chamber, or when it flowed through a commercial microfluidic slide (Luer). In fact, the device presented the following advantages: simple and low-cost production, integrability into the Raman probe system, stability of the results during the measurement process and ease of automation of the process, allowing its use for continuous monitoring by non-highly trained personnel.

## Figures and Tables

**Table 1:** Values associated with the dimensions labelled in Figure 2 corresponding to the through channel glass design, top cover glass and glass-to-tube adapters. Units are in millimetres.

Global	Radii	Centres	Lengths	Widths	Diameters *	Heights *
W = 20	R_*in*_ = 6.90	$C_{1}\left(0,1\right)$	L_1_ = 5.0	w_1_ = 0.20	d_1_ = 1.1	h_1_ = 8.7
L = 20	R_*out*_ = 7.10	$C_{2}\left(-7,-7\right)$	L_2_ = 2.0	w_2_= 0.65	d_2_ = 1.3	h_2_ = 8.4
d = 1	R = 0.75	$C_{3}\left(7,-7\right)$	L_3_ = 1.0		d_3_ = 3.2	h_3_ = 1.0
					d_4_ = 5.0	

* Diameters and heights of the adapters.

**Table 2:** Measurement procedure and inverted time and effort comparison between substrates during microfluidic device validation.

	20 µL Well	5 µL Well	Counting Chamber	Luer	Microfluidic Device
Preparation of the sample	Pipette sample on substrate			Introduce tubes on containers	
Initialization of the measurement	Delimitation of polygonal region for mapping			Focalize point for measurement	
Time measuring	30 min	40 min	20 min	30 min	6 min

## Data Availability

Data availability can be requested through the corresponding author.

## Abbreviations

The following abbreviations are used in this manuscript:

HABs	Harmful Algal Blooms
SEM	Scanning Electron Microscopy
TEM	Transmission Electron Microscopy
FISH	Fluorescence in situ hybridization
PCR	Polymerase Chain Reaction
ELISA	Enzyme-Linked Immunosorbent Assay
RRS	Resonance Raman spectroscopy
SERS	Surface-Enhanced Raman spectroscopy
TERS	Tip-Enhanced Raman spectroscopy
CAD	Computer-Aided Design
UV	Ultraviolet
ULAE	Ultrafast laser-assisted etching
FLCPA	Femtosecond Laser Chirped Pulse Amplifier
CCD	Charge-Coupled Device
HF	Hydrofluoric acid
NaOH	Sodium hydroxide
PRR	Pulse Repetition Rate
ROI	Region Of Interest
LOD	Limit of Detection
